# Supplementary material for: Association between first-line antidepressant use and risk of dementia in older adults: a retrospective cohort study
Source: BMC Geriatr. 2023 Dec 8;23:825. doi: 10.1186/s12877-023-04475-z (PMC10709864; doi:10.1186/s12877-023-04475-z)
Supplement: Supplementary file 1 — Additional file 1: Appendix Figure 1. Directed acyclic graph illustrating the relationships among exposure, outcome, and confounders. Appendix Figure 2. Detailed steps of the statistical analysis. Appendix Table 1. Variables included in our study. [file 12877_2023_4475_MOESM1_ESM.docx]

**APPENDIX**

**Appendix Figure 1.** Directed acyclic graph illustrating the relationships among exposure, outcome, and confounders.

**Appendix Figure 2.** Detailed steps of the statistical analysis.

**Appendix Table 1.** Variables included in our study.

**
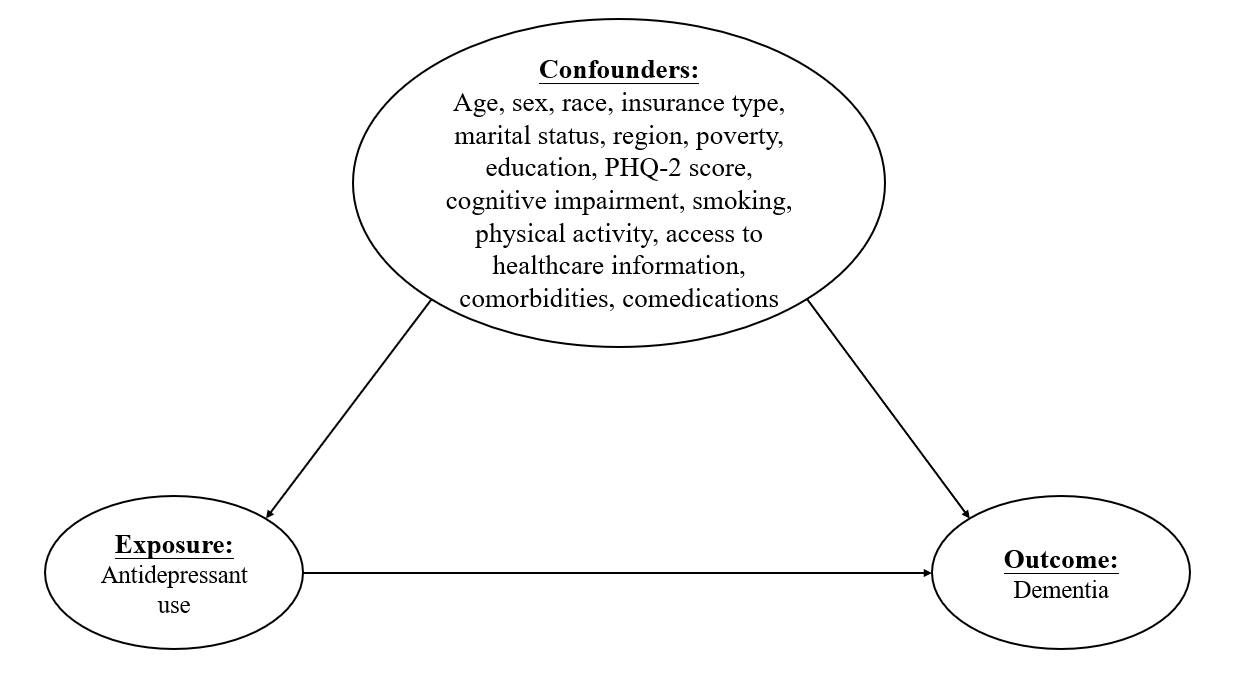
**

**Appendix Figure 1. Directed acyclic graph illustrating the relationships among exposure, outcome, and confounders.**


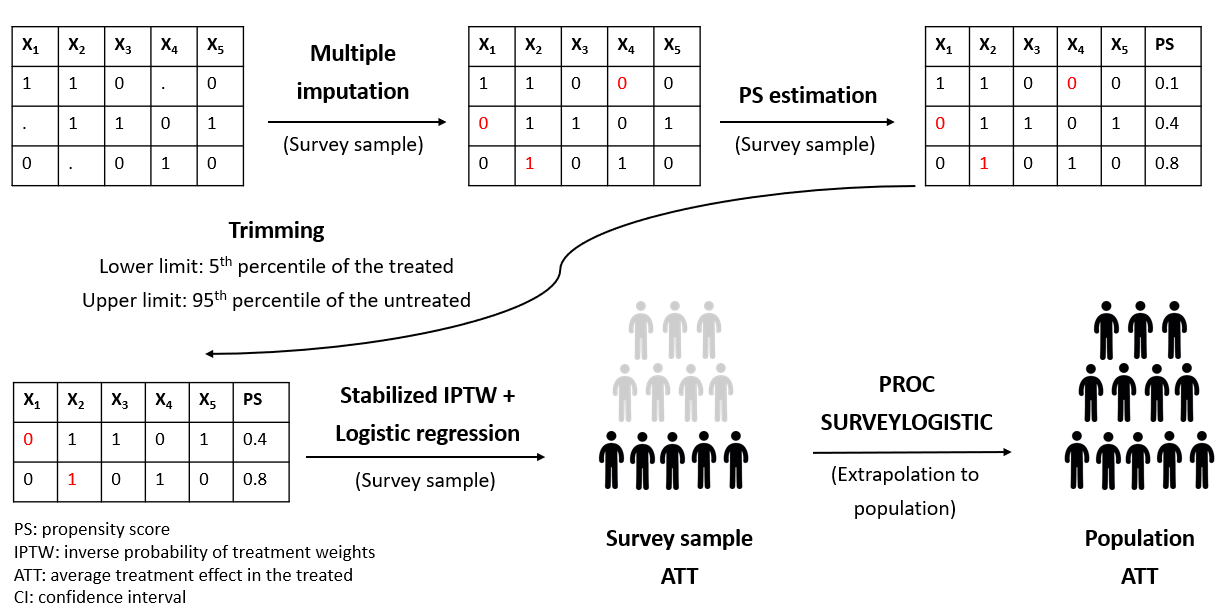


**Appendix Figure 2. Detailed steps of the statistical analysis.**

**Appendix Table 1. Data sources and operational definitions of the variables included in the study**

| Variable | MEPS File name | MEPS Variable name | Operational definition or codes for the study variables |
| --- | --- | --- | --- |
| **Study population** | | | |
| Depression | Medical Conditions file | ICD9CODX | 296.20-296.25, 296.30-296.35, 300.4, 311  PPV: 92%(15) |
|  |  | ICD10CDX | F32.0-32.9, F33.0-33.3, F33.8, F33.9, F34.1 & F41.2  PPV: 91.06% |
| PHQ-2 | Full-Year Consolidated File | PHQ242 | PHQ-2 score ranges from 0-6. The authors identified a score of 3 as the optimal cutpoint when using the PHQ-2 to screen for depression.  If the score is 2 or greater, major depressive disorder is likely (sensitivity: 86% and specificity: 78%)(17) |
| **Exposure/Comparison** | | | |
| SSRIs | Prescribed medicines file | TC1S1_1, TC2S1_1 | 208 |
| SNRIs | Prescribed medicines file | TC1S1_1, TC2S1_1 | 308 |
| Psychotherapy | Outpatient Visits | VSTCTGRY | 4 |
|  | Office-Based Medical Provider Visits | VSTCTGRY | 4 |
| **Outcome** | | | |
| Dementia | Medical Conditions file | ICD9CODX | 290, 331.0, 331.1, 331.2, 331.82, 331.83, 331.9, 438.0, 780.93  PPV: 73.2-93.6%(15) |
|  |  | ICD10CDX | F00, F01, F03, F04, G30, G31.0, G31.1, G31.8, G31.9, I69.91, R41  PPV: 73.2-93.6%(15) |
| Cognitive impairment | Full-Year Consolidated File | COGLIM31, COGLIM53, DFCOG42 | COGLIM31, COGLIM53: cognitive limitations  DFCOG42: serious cognitive difficulties  Both are yes/no questions |
| **Covariates** | | | |
| Age | Full-Year Consolidated File | AGE31X | -1: inapplicable  Continuous variable |
| Sex | Full-Year Consolidated File | SEX | 1: male (ref)  2: female |
| Race | Full-year Consolidated File | RACEV1X | 1: White – No other race reported (ref)  2: Black – No other race reported  3: AI/AN - No other race  4: Asian/Native Hawaiian/Pacific Islander  6: Multiple races reported |
| Region | Full-Year Consolidated File | REGION31 | Census region  1: Northeast (ref)  2: Midwest  3: South  4: West |
| Education | Full-Year Consolidated File | HIDEG | Highest degree when first entered MEPS  1: No degree  2: General education development (ref)  3: High school diploma  4: Bachelor’s degree  5: Master’s degree  6: Doctorate degree  7: Other degree  8: Under 16 - inapplicable |
| Type of insurance | Full-Year Consolidated File | INSCOV11 | 1: Any private  2: Public only  3: Uninsured |
| Poverty | Full-Year Consolidated File | POVCAT10  POVCAT11  POVCAT12  ... | Family income as % of poverty line  1: poor/negative (less than 100% (poverty line)) (ref)  2: near poor (100% to less than 125%)  3: low income (125% to less than 200%)  4: middle income (200% to less than 400%)  5: high income (>=400%) |
| Marital status | Full-year Consolidated File | MARRY31X | 1: married (ref)  2: widowed  3: divorced  4: separated  5: never married  6: under age 16 – inapplicable  7: married in round  8: widowed in round  9: divorced in round  10: separated in round |
| Physical inactivity | Full-Year Consolidated File | WLKLIM31 | Limitation in physical functioning  1: Yes  2: No (ref) |
| Smoking | Full-Year Consolidated File | ADSMOK42 | Currently smoke  -9: not ascertained  -1: inapplicable  1: yes  2: no (ref) |
| Access to care | Full-year Consolidated File | MDDLAY42 | Delayed in getting necessary medical care  -9: not ascertained  -8: DK  -7: refused  -1: inapplicable  1: yes  2: no (ref) |
|  |  | MDUNAB42 | Unable to get necessary medical care  -9: not ascertained  -8: DK  -7: refused  -1: inapplicable  1: yes  2: no (ref) |
|  |  | PMDLAY42 | Delayed in getting necessary prescribed medications  -9: not ascertained  -8: DK  -7: refused  -1: inapplicable  1: yes  2: no (ref) |
|  |  | PMUNRS42 | Unable to get necessary prescribed medications  -9: not ascertained  -8: DK  -7: refused  -1: inapplicable  1: yes  2: no (ref) |
| **Comorbidities** | | | |
| Cancer | Medical Conditions file | ICD9CODX | 140-209 |
|  |  | ICD10CODX | C00-C96 |
| Type 2 diabetes |  | ICD9CODX | 250 |
|  |  | ICD10CODX | E11 |
| Hyperlipidemia |  | ICD9CODX | 272 |
|  |  | ICD10CODX | E78 |
| Hypertension |  | ICD9CODX | 401-405 |
|  |  | ICD10CODX | I10-I16 |
| Ischemic stroke |  | ICD9CODX | 433-437 |
|  |  | ICD10CODX | I63-I68 |
| Coronary heart disease |  | ICD9CODX | 410-414 |
|  |  | ICD10CODX | I20-I25 |
| Osteoarthritis |  | ICD9CODX | 715 |
|  |  | ICD10CODX | M15-M19 |
| Parkinson’s disease |  | ICD9CODX | 332.0 |
|  |  | ICD10CODX | G20 |
| Anxiety |  | ICD9CODX | 300.0, 300.2, 300.3, 309.8 |
|  |  | ICD10CODX | F40, F41, F42, F93.0–F93.2 |
| Sleep disorder |  | ICD9CODX | 307.4, 780.5 |
|  |  | ICD10CODX | G47.8, G47.9 |
| Schizophrenia |  | ICD9CODX | 295.0–295.9 |
|  |  | ICD10CODX | F20–F29 |
| Bipolar disorder |  | ICD9CODX | 296.0–296.1, 296.4–296.8 |
|  |  | ICD10CODX | F30, F31, F34.0 |
| **Comedications** | | | |
| Analgesics | Prescribed medicines file | TC1S1 | 58 |
| Benzodiazepines |  | TC1S1 | 69 |
| Anxiolytics, sedatives, and hypnotics |  | TC1S1 | 67 |
| Antidepressants other than SSRI/SNRI |  | TC1S1_1 | 76, 209, 306, 307 |
| Antipsychotics |  | TC1S1  TC2S1 | 251 |
| Antiparkinsonian agents |  | TC1S1 | 66 |

*Abbreviations: PHQ, Patient Health Questionnaire; SSRI, selective serotonin reuptake inhibitors; SNRI, serotonin and norepinephrine reuptake inhibitors*
